# Supplementary material for: Quantum algorithms for equational reasoning
Source: Sci Adv. 2026 May 15;12(20):eaec2736. doi: 10.1126/sciadv.aec2736 (PMC13178570; doi:10.1126/sciadv.aec2736)
Supplement: Supplementary file 1 — Supplementary Text Figs. S1 and S2 References [file sciadv.aec2736_sm.pdf]

Science Advances
AAAS

Supplementary Materials for

Quantum algorithms for equational reasoning

Davide Rattacaso *et al.*

Corresponding author: Davide Rattacaso, davide.rattacaso@unipd.it

Sci. Adv. **12**, eaec2736 (2026)
DOI: 10.1126/sciadv.aec2736

This PDF file includes:

Supplementary Text
Figs. S1 and S2
References

Supplementary Text

Simulating the Hamiltonian

Here, we analyze the quantum resources required for simulating the Hamiltonian $\hat{H}_{S,\tilde{\omega}}$, specifically in terms of the number of quantum gates.

We do not consider native qudit platforms (*78*), which might eventually prove advantageous for operating with rewriting systems whose alphabet contains more than two elements. Instead, we focus on standard qubit-based computation. To this end, we note that any rewriting system defined on an alphabet of size d can be translated into a rewriting system over a binary alphabet, as happens when strings are manipulated in standard (Boolean) classical computers. In particular, each symbol of the original alphabet can be encoded as a binary string of length $\lceil \log_2 d \rceil$, i.e., its binary logarithm rounded to the nearest bigger integer. This corresponds to the binary representation of the symbol index. For example, for the alphabet $A = \{a, b, c\}$, we can assign: $a \rightarrow (0, 0)$, $b \rightarrow (0, 1)$, and $c \rightarrow (1, 0)$. Similarly, a string of L characters over A is mapped to a binary string of length $L \cdot \lceil \log_2 d \rceil$. Each rewriting rule of length l is likewise mapped to a rule of length $l \cdot \lceil \log_2 d \rceil$, while the total number n_r of rewriting rules remains unchanged.

With this binary encoding in place, we can restrict our analysis to simulating $\hat{H}_{S,\tilde{\omega}}$ over the binary alphabet $A = \{0, 1\}$, which is compatible with standard qubit-based quantum devices.

The simulation typically relies on a discretization of the time evolution, allowing the approximation of the evolution operator as a product of exponentials of the individual Hamiltonian terms (*79*). The Trotterized evolution, or any analogous optimization ansatz such as QAOA, can be implemented by the application of the operator

$$U = \prod_{i}^{N} \left(\mathrm{e}^{i\alpha_i |\tilde{\omega}\rangle\langle\tilde{\omega}|} \prod_{r \in R} \mathrm{e}^{-i\beta_{r,i}\hat{r}^2} \mathrm{e}^{i\beta_{r,i}\hat{r}} \right) , \tag{S1}$$

where the α_i and β_i are real coefficients scaling as the time step $\delta_\tau = \tau/N$, the indices i run over the N time steps, and τ is the total evolution time. The operator consists of one term involving the exponential of the projector $|\tilde{\omega}\rangle\langle\tilde{\omega}|$, and a sequence of terms derived from the Laplacian. The number of Laplacian-related operators is twice the number n_r of rewriting rules in the system, due to the squared and linear terms for each rule.

Thus, the entire evolution can be realized by implementing as a circuit consisting of $N \cdot (2n_r + 1)$

operators

$$W = \mathrm{e}^{-i\theta|\mathbf{b}''\rangle\langle\mathbf{b}'|}\,, \tag{S2}$$

where $\mathbf{b}'$ and $\mathbf{b}''$ are binary substrings of size l (for operators encoding rules) or L (for the operator encoding the projection on the word $\tilde{\omega}$), and N is the total number of time steps.

The operators in Eq. (S2) can be implemented in a quantum circuit (see Figure S1). First, we use a combination of X gates and a multi-controlled NOT gate to flip an ancilla qubit, conditioned on the local quantum state matching $|\mathbf{b}'\rangle$. Next, we apply two-qubit gates with the control qubit being the ancilla to transform the state $|\mathbf{b}'\rangle$ into $\mathrm{e}^{-i\theta}|\mathbf{b}''\rangle$. Finally, we reverse the ancilla operation by applying the same X gates and multi-controlled NOT gate, effectively uncomputing the ancilla. Thus, when the operator W to be implemented corresponds to a rewriting rule r, it requires $O(w_r)$ gates, where w_r denotes the number of characters the rule acts upon. The implementation involves two multi-controlled gates. Similarly, if W represents the evolution generated by the projection onto the input state, it requires $O(L)$ gates, with L being the system size. In this case as well, only two multi-controlled gates are needed. Multi-controlled gates can be implemented efficiently on universal quantum computers using a linear number of elementary gates and ancilla qubits (*73*). Alternatively, they may be natively supported on certain hardware platforms, such as Rydberg-atom arrays and superconducting circuits (*74*). In both scenarios, implementing the operator W requires a number of long-range two-qubit gates that scales linearly with the length of the binary string $\mathbf{b}'$.

Overall, simulating the Trotterized evolution over N steps requires

$$D = O(N(2w \cdot n_r + L)) \tag{S3}$$

gates, where w is the maximum number of characters affected by any rule in the rewriting system. These resources are linear in the number of bits needed to describe the problem instance classically—that is, the input string and the set of rewriting rules.

As a consequence of the Baker–Campbell–Hausdorff formula, the first-order Trotterized simulation in Eq. (S1) incurs, at each time step, a local error scaling as $\delta_\tau^2 = O(\tau^2/N^2)$. Higher-order Trotter–Suzuki decompositions can be employed to systematically reduce this error. Restricting here to the first-order approximation, the cumulative error over the full time evolution scales as $O(\tau^2/N)$. Therefore, in order to approximate the continuous-time dynamics within a target Trotter error ϵ, one requires $N = O(\tau^2/\epsilon)$ discrete time steps.

When the objective is ground-state preparation, and assuming a simple adiabatic evolution as the heuristic method of choice, the total evolution time required to reach the target ground state is expected to scale as $\Delta_{\min}^{-2}$ (*32*), where $\Delta_{\min}$ denotes the minimum energy gap of the Hamiltonian restricted to the dynamically accessible subspace. This subspace is spanned by computational basis states that are equivalent under the rewriting rules, to which the dynamics is constrained by construction. Combining the adiabatic time scaling with the first-order Trotterization error bound yields a total number of Trotter steps scaling as $N = \mathcal{O}(\Delta_{\min}^{-4})$. The resulting circuit depth can thus be bounded by

$$D = \mathcal{O}\left(\Delta_{\min}^{-4}\,(2w \cdot n_r + L)\right) . \tag{S4}$$

This scaling can be easily improved through a variety of techniques, including higher-order Trotterization schemes, counterdiabatic driving and other shortcuts to adiabaticity, as well as heuristics better suited to near-term quantum hardware, such as the Quantum Approximate Optimization Algorithm.

Fidelity between orbit states on quantum annealers

Here, we show how the fidelity between orbit states can be measured on quantum annealers.
We prepare the orbit states $|X_{S',\omega'}\rangle$ and $|X_{S'',\omega''}\rangle$ on a quantum annealer. To this aim, we initialize the annealer respectively in the states $|\omega'\rangle$ and $|\omega''\rangle$. We evolve these initial states with the Hamiltonians $H_{S',\omega'}(t)$ and $H_{S'',\omega''}(t)$ for large enough times τ' and τ''. In the adiabatic regime, we obtain

$$|X_{S',\omega'}\rangle = \mathcal{T}\left[\mathrm{e}^{-i\int_0^{\tau'} dt H_{S',\omega'}(t)}\right]|\omega'\rangle , \tag{S5}$$

and

$$|X_{S'',\omega''}\rangle = \mathcal{T}\left[\mathrm{e}^{-i\int_0^{\tau''} dt H_{S'',\omega''}(t)}\right]|\omega''\rangle , \tag{S6}$$

where $\mathcal{T}$ is the time-ordering operator.

Considering that $\mathcal{T}\left[\mathrm{e}^{-i\int_0^{\tau'} dt H_{S',\omega'}(t)}\right]^\dagger = \mathcal{T}\left[\mathrm{e}^{-i\int_0^{\tau'} dt H_{S',\omega'}(\tau'-t)}\right]$, the fidelity between the orbit states is

$$|\langle X_{S'',\omega''}|X_{S',\omega'}\rangle|^2 = \left|\langle\omega'|\mathcal{T}\left[\mathrm{e}^{-i\int_0^{\tau'} dt H_{S',\omega'}(\tau'-t)}\right] \cdot \right.$$
$$\left. \cdot\,\mathcal{T}\left[\mathrm{e}^{-i\int_0^{\tau''} dt H_{S'',\omega''}(t)}\right]|\omega''\rangle\right|^2 . \tag{S7}$$

Now, we define the state

$$|\omega''_{S',S''}\rangle = \mathcal{T}\left[\mathrm{e}^{-i\int_0^{\tau'} dt H_{S',\omega'}(\tau'-t)}\right] \cdot \mathcal{T}\left[\mathrm{e}^{-i\int_0^{\tau''} dt H_{S'',\omega''}(t)}\right]|\omega''\rangle \ . \tag{S8}$$

This state can be prepared on the quantum annealer by evolving the initial state $|\omega''\rangle$ first with Hamiltonian $H_{S'',\omega''}(t)$ for a time τ'', and then with the Hamiltonian $H_{S',\omega'}(\tau'-t)$ for a time τ'.

Once $|\omega''_{S',S''}\rangle$ has been prepared, the fidelity in Eq. (S7) is measured as the expectation value of the projector $P_{\omega'} = |\omega'\rangle\langle\omega'|$ onto the computational basis state $|\omega'\rangle$, i.e.,

$$|\langle X_{S'',\omega''}|X_{S',\omega'}\rangle|^2 = \langle\omega''_{S',S''}|P_{\omega'}|\omega''_{S',S''}\rangle \ . \tag{S9}$$

This quantity is the probability of sampling ω'. It can be estimated by performing N_s shots of the experiment and counting the frequency of outcomes corresponding to the configuration ω'. The error on this estimation scales as $O(1/\sqrt{N_s})$.

Comparison to classical approaches

Here, we analyze connections and differences between quantum normal form reduction and the main classical approaches to the word problem and the counting problem. This comparison clarifies in which limits quantum normal form reduction can be regarded as a quantum extension of classical methods, and how insights from these approaches may be leveraged to improve quantum normal form reduction.

Graph exploration

The most direct classical approach is based on explicit graph-exploration algorithms, such as breadth-first search with memoization (*67*). These algorithms sequentially explore the connected component of the rewriting graph containing the input string, allowing one to test equivalence and to enumerate or count all connected words. Since the size of the connected subgraph typically grows exponentially with the size of the rewriting system or the input, such approaches quickly become computationally infeasible.

Adiabatically preparing the ground state of the Laplacian associated with the rewriting system corresponds to exploring the graph of connected words, but in quantum superposition rather than

via sequential traversal. From this perspective, quantum normal form reduction is more naturally compared to random walks on graphs, whose equilibrium distribution is uniform over connected components. In both the classical random-walk and the quantum settings, the computational complexity is governed by the bottlenecks of the graph, which determine the spectral gap of the Laplacian. Overall, the possibility of a quadratic quantum speedup relative to classical random walks has been extensively studied in the literature, although its realization depends sensitively on structural properties of the specific rewriting system (*80*).

Canonical form reduction

More sophisticated approaches to the word problem are based on canonical form reduction (*2*), that is, on constructing a procedure that maps all elements of the same equivalence class to a unique representative. This allows one to solve the word problem by comparing the normal forms associated with the input words. Quantum normal form reduction can be viewed as a quantum analogue of this paradigm, since it maps all equivalent strings to a single coherent quantum superposition. Unlike classical canonical forms, this superposition can address the counting problem, since it encodes information about the entire equivalence class.

Completion-based methods, most notably the Knuth–Bendix algorithm (*27*), provide a systematic way to obtain such canonical forms by transforming the original rewriting system into one that is both terminating and confluent. Completion can fail or not terminate, limiting the applicability of this approach. In contrast to quantum normal form reduction, the Knuth–Bendix procedure requires the user to specify a suitable reduction ordering, and its success is highly sensitive to this choice. In Section *Knuth-Bendix algorithm* of this Supplementary Text, we compare the performance of the Knuth–Bendix algorithm with our approach for a specific case.

Automata-based methods

Automata-based methods consist of constructing an accepting automaton, i.e., a finite-state automaton that accepts exactly the strings connected from a given input, thereby solving the word problem (*12*). Since each accepting path through the automaton corresponds to a distinct string, dynamic programming techniques can be used to count connected words.

The tensor-network representation of orbit states provides a bridge between quantum normal form reduction and automata-based constructions. Indeed, matrix product states are equivalent to weighted finite-state automata that compute functions on strings, where the bond dimension controls

the number of internal automaton configurations. Thus, orbit states can be interpreted as accepting automata with exponentially large expressive power, since a generic quantum state corresponds to a matrix product state with a bond dimension exponential in the system size. Quantum normal form reduction defines a systematic procedure for implicitly constructing such automata.

Reduction to SAT

Boolean satisfiability (SAT)–based methods typically encode the predicate "ω_1 rewrites to ω_2 within k rewrite steps" as a Boolean formula that is satisfiable if and only if such a derivation exists (*81*). This encoding enables the use of highly optimized SAT solvers to address instances of the word problem. Furthermore, reductions to SAT allow one to enumerate satisfying assignments using blocking clauses, although counting an exponentially large set of connected strings in this way generally requires an exponential number of solver calls. Approximate counting can be achieved with a polynomial number of SAT solver invocations using hashing-based techniques. These methods estimate the number of satisfying assignments of a Boolean formula by randomly partitioning the solution space in buckets using XOR constraints, and then exactly counting the solutions in a randomly selected bucket whose expected size is bounded by a fixed threshold (*82*).

Since the Laplacian can be written as a frustration-free sum of positive semi-definite operators $r^2 - r$, preparing orbit states is a quantum analogue of SAT-based approaches, but with non-commuting clauses (*83*). While the annealing-based scheme proposed here should therefore be regarded as a heuristic that may be effective in typical instances, exploring connections with modern SAT solver heuristics could improve this approach.

Unlike the solution of a SAT encoding, the ground state of the Laplacian is unique and naturally encodes a coherent superposition of all classical solutions. Estimating the number of connected strings nevertheless still requires repeated runs of the algorithm to sample the overlap between the orbit state and the uniform superposition over connected strings (*43*). An analogue of approximate counting would be to restrict the Hilbert space to randomly chosen buckets, implemented by adding suitable penalty terms to the Laplacian operator.

Knuth-Bendix algorithm

The Knuth-Bendix algorithm (*27*) is a state-of-the-art classical approach for solving the word problem. This procedure transforms the original rewriting system S into a new and non-invertible

system S_C, whose rules increase a specified total order on strings, such as lexicographic order. The new system is equivalent to the original one, meaning that two strings are connected by S if and only if they are connected by S_C. Moreover, the transformed system S_C is both *terminating*, meaning that no infinite sequence of rule applications is possible, and *confluent*, meaning that any sequence of valid rule applications yields the same final result, regardless of the order in which rules are applied.

For a rewriting system that is both confluent and terminating, two strings ω' and ω'' are equivalent if and only if repeated application of rewriting rules — regardless of the order in which they are applied — reduces both to the same string. This unique representative is called the *normal form*. Reduction to normal form thus enables efficient resolution of the word problem, provided that such a system S_C can be constructed.

The Knuth-Bendix algorithm is not guaranteed to terminate: in general, the construction of S_C may fail, reflecting the undecidability of the word problem for arbitrary rewriting systems. However, a fair comparison with the Knuth-Bendix algorithm for the purposes of this work must account for the finite size of the strings. This constraint limits the maximum length of the rewriting rules generated during the execution of the algorithm, ensuring termination within finite time and memory resources that depend on L.

As a benchmark for our quantum algorithm, we use the computer algebra system *GAP* (*75*) to run the Knuth-Bendix algorithm for the string rewriting system in Eq. (29). The algorithm is executed for both the *shortlex* and *recursive* orderings, and for both possible permutations of the alphabet, (a, b) and (b, a) (see GAP documentation for further details). All executions were performed on a virtual machine equipped with 6 Intel(R) Core(TM) i5-8500 CPUs and 16 GB of memory. The total execution time until termination, as well as the memory footprint of the resulting confluent rewriting system S_C, are reported in Figure S2 for string lengths up to $L = 400$. Across different choices of ordering, the computational time scales asymptotically as $O(L^{\sim 6.4})$. The number of rules in S_C grows as $O(L^{\sim 2.1})$, while the total memory required to store the system — measured by the cumulative length of all rules — scales as $O(L^{\sim 3.0})$.

Computational complexity of the imaginary quantum annealing

Here, we bound the computational complexity of imaginary quantum annealing (IQA) with respect to the energy gap and the fidelity susceptibility of the driving Hamiltonian ground state.

As in the main text, we consider IQA discretized in N steps. We fix the time duration δ_τ of each step, so that the total annealing time is $\tau = \delta_\tau N$.

The system's Hamiltonian at the step s is

$$\hat{H}_s = \left[s\delta \hat{\mathcal{L}}_S - (1 - s\delta) \left|\tilde{\omega}\right\rangle\left\langle\tilde{\omega}\right| \right] , \tag{S10}$$

where $\delta = 1/N$ is the variation of the Hamiltonian parameter per step.

We call $|\psi_s\rangle$ the state of the system at step $s \in [0, \ldots, N]$, $|0_s\rangle$ the ground state of the Hamiltonian $\hat{H}_s$, and $E_{n,s}$ the n-th energy level of $\hat{H}_s$.

We measure the error in approximating the ground state at a step s as the infidelity $1 - F_s$ between $|\psi_s\rangle$ and $|0_s\rangle$, where the fidelity F_s is defined as

$$F_s = |\langle 0_s|\psi_s\rangle|^2 \ . \tag{S11}$$

The state evolution at each step is

$$|\psi_{s+1}\rangle = \frac{\mathrm{e}^{-H_{s+1}\delta_\tau}|\psi_s\rangle}{\left\| \mathrm{e}^{-H_{s+1}\delta_\tau}|\psi_s\rangle \right\|} , \tag{S12}$$

so that the fidelity is

$$|\langle 0_{s+1}|\psi_{s+1}\rangle|^2 = \frac{\left|\langle 0_{s+1}|\mathrm{e}^{-H_{s+1}\delta_\tau}|\psi_s\rangle\right|^2}{\langle\psi_s|\mathrm{e}^{-2H_{s+1}\delta_\tau}|\psi_s\rangle} \ . \tag{S13}$$

The numerator of the last equation can be written as

$$\left|\langle 0_{s+1}|\mathrm{e}^{-H_{s+1}\delta_\tau}|\psi_s\rangle\right|^2 = \mathrm{e}^{-2E_{0,s+1}\delta_\tau}\left|\langle 0_{s+1}|\psi_s\rangle\right|^2 \ . \tag{S14}$$

Let $P_s = |0_s\rangle\langle 0_s|$ be the projector on the ground state $|0_s\rangle$, and $P_s^\perp = 1 - |0_s\rangle\langle 0_s|$ its orthogonal complement. We have:

$$\mathrm{e}^{-H_{s+1}\delta_\tau}|\psi_s\rangle = \mathrm{e}^{-H_{s+1}\delta_\tau}\left(P_{s+1} + P_{s+1}^\perp\right)|\psi_s\rangle , \tag{S15}$$

so that the denominator becomes

$$\begin{aligned}\left\|\mathrm{e}^{-H_{s+1}\delta_\tau}|\psi_s\rangle\right\|^2 &= \langle\psi_s|\left(P_{s+1}+P_{s+1}^\perp\right)\mathrm{e}^{-2H_{s+1}\delta_\tau}\left(P_{s+1}+P_{s+1}^\perp\right)|\psi_s\rangle\\ &= \langle\psi_s|P_{s+1}\mathrm{e}^{-2H_{s+1}\delta_\tau}P_{s+1}|\psi_s\rangle + \langle\psi_s|P_{s+1}^\perp\mathrm{e}^{-2H_{s+1}\delta_\tau}P_{s+1}^\perp|\psi_s\rangle\\ &\le \mathrm{e}^{-2E_{0,s+1}\delta_\tau}\langle\psi_s|P_{s+1}|\psi_s\rangle + \mathrm{e}^{-2E_{1,s+1}\delta_\tau}\langle\psi_s|P_{s+1}^\perp|\psi_s\rangle\\ &= \mathrm{e}^{-2E_{0,s+1}\delta_\tau}\left|\langle 0_{s+1}|\psi_s\rangle\right|^2 + \mathrm{e}^{-2E_{1,s+1}\delta_\tau}\left(1-\left|\langle 0_{s+1}|\psi_s\rangle\right|^2\right)\,, \end{aligned}\tag{S16}$$

where at the second line we exploited the equation $P_{s+1}H_{s+1}P_{s+1}^\perp = 0$, and, at the third line, we consider that $\langle\psi_s|P_{s+1}\mathrm{e}^{-2H_{s+1}\delta_\tau}P_{s+1}|\psi_s\rangle = \langle\psi_s|P_{s+1}|\psi_s\rangle\mathrm{e}^{-2E_{0,s+1}\delta_\tau}$ and $\langle\psi_s|P_{s+1}^\perp\mathrm{e}^{-2H_{s+1}\delta_\tau}P_{s+1}^\perp|\psi_s\rangle \le \langle\psi_s|P_{s+1}^\perp|\psi_s\rangle||P_{s+1}^\perp\mathrm{e}^{-2H_{s+1}\delta_\tau}P_{s+1}^\perp||_{\mathrm{op}} \le \langle\psi_s|P_{s+1}^\perp|\psi_s\rangle\mathrm{e}^{-2E_{1,s+1}\delta_\tau}$, where $||A||_{\mathrm{op}}$ is the operator norm of A.

Substituting the corresponding terms in Eq. (S13) with the results of Eq. (S14) and Eq. (S16), we obtain

$$\begin{aligned}F_{s+1} &\ge \frac{\mathrm{e}^{-2E_{0,s+1}\delta_\tau}\left|\langle 0_{s+1}|\psi_s\rangle\right|^2}{\mathrm{e}^{-2E_{0,s+1}\delta_\tau}\left|\langle 0_{s+1}|\psi_s\rangle\right|^2 + \mathrm{e}^{-2E_{1,s+1}\delta_\tau}\left(1-\left|\langle 0_{s+1}|\psi_s\rangle\right|^2\right)}\\ &= \frac{\left|\langle 0_{s+1}|\psi_s\rangle\right|^2}{\left|\langle 0_{s+1}|\psi_s\rangle\right|^2 + \mathrm{e}^{-2\Delta_{s+1}\delta_\tau}\left(1-\left|\langle 0_{s+1}|\psi_s\rangle\right|^2\right)}\,,\end{aligned}\tag{S17}$$

where Δ_{s+1} is the first energy gap of $\hat{H}_{s+1}$.

Thus, the infidelity can be bounded as

$$\begin{aligned}1-F_{s+1} &\le \frac{\mathrm{e}^{-2\Delta_{s+1}\delta_\tau}\left(1-\left|\langle 0_{s+1}|\psi_s\rangle\right|^2\right)}{\left|\langle 0_{s+1}|\psi_s\rangle\right|^2 + \mathrm{e}^{-2\Delta_{s+1}\delta_\tau}\left(1-\left|\langle 0_{s+1}|\psi_s\rangle\right|^2\right)}\\ &\le \mathrm{e}^{-2\Delta_{s+1}\delta_\tau}\frac{1-\left|\langle 0_{s+1}|\psi_s\rangle\right|^2}{\left|\langle 0_{s+1}|\psi_s\rangle\right|^2}\,.\end{aligned}\tag{S18}$$

Now, we want to relate the infidelity at the step $s+1$ to the infidelity at the step s.
First, we decompose $|\psi_s\rangle$ on its parallel and orthogonal component with respect to $|0_s\rangle$, that is, $|\psi_s\rangle = \sqrt{F_s}|0_s\rangle + \mathrm{e}^{i\theta_s}\sqrt{1-F_s}|e_s\rangle$ for some complex phase θ_s. Thus we have

$$\langle 0_{s+1}|\psi_s\rangle = \sqrt{F_s}\langle 0_{s+1}|0_s\rangle + \mathrm{e}^{i\theta_s}\sqrt{1-F_s}\langle 0_{s+1}|e_s\rangle\,.\tag{S19}$$

At this point, we introduce the fidelity susceptibility f_s for the ground state path of H_s as a measure of the infinitesimal variation of the ground state (*84*), i.e.:

$$|\langle 0_{s+1}|0_s\rangle|^2 = 1 - f_s\delta^2\,.\tag{S20}$$

which implies

$$|0_{s+1}\rangle = \sqrt{1-f_s\delta^2}|0_s\rangle + \sqrt{f_s}\delta \mathrm{e}^{i\theta'_s}|\varepsilon_s\rangle \ , \tag{S21}$$

where $|\varepsilon\rangle$ is the orthogonal part of $|0_{s+1}\rangle$ with respect to $|0_s\rangle$ and θ'_s is a complex phase.

Equation (S21) holds whenever the ground state path $|0_s\rangle$ is derivable. Being the Hamiltonian H_s derivable, this happens whenever there is no level crossing between the first and the second energy level. In the case under exam here, where H_s has negative non-diagonal entries, the Perron–Frobenius theorem guarantees the uniqueness of the ground state at each s and therefore the absence of level crossing. Considering together Eq. (S19) and Eq. (S21) we obtain

$$|\langle 0_{s+1}|\psi_s\rangle|^2 = \left|\sqrt{F_s}\sqrt{1-f_s\delta^2} + \mathrm{e}^{i\theta_s}\mathrm{e}^{i\theta'_s}\sqrt{1-F_s}\sqrt{f_s}\delta\langle e_s|e_s\rangle\right|^2 \ . \tag{S22}$$

Exploiting the triangular inequality and considering that for small enough δ, that is for large N, the modulus of the second addend is smaller than the modulus of the first, we obtain

$$|\langle 0_{s+1}|\psi_s\rangle|^2 \geq \left|\sqrt{F_s}\sqrt{1-f_s\delta^2} - \sqrt{1-F_s}\sqrt{f_s}\delta|\langle e_s|e_s\rangle|\right|^2 \geq F_s(1-f_s\delta^2) \ . \tag{S23}$$

Now we substitute Eq. (S23) in Eq. (S18) to obtain the relationship between fidelity at successive steps for large N:

$$1 - F_{s+1} \leq \mathrm{e}^{-2\Delta_{s+1}\delta_\tau}\frac{1-F_s(1-f_s\delta^2)}{F_s(1-f_s\delta^2)} \approx \mathrm{e}^{-2\Delta_{s+1}\delta_\tau}\left(\frac{1-F_s}{F_s} + \frac{f_s}{F_s^2}\delta^2\right) \tag{S24}$$

Finally, we iteratively apply the last equation to obtain the evolution of the infidelity. Considering that at the step $s = 0$ the system is in the exact ground state of the Hamiltonian, so that $F_0 = 1$, we have

$$1 - F_1 \leq \mathrm{e}^{-2\Delta_1\delta_\tau} f_0\delta^2 \ . \tag{S25}$$

and for $s = 2$, considering small δ, we have

$$\begin{aligned} 1 - F_2 &\leq \mathrm{e}^{-2\Delta_2\delta_\tau}\left(\frac{\mathrm{e}^{-2\Delta_1\delta_\tau} f_0\delta^2}{1-\mathrm{e}^{-2\Delta_1\delta_\tau} f_0\delta^2} + \frac{f_1}{\left(1-\mathrm{e}^{-2\Delta_1\delta_\tau} f_0\delta^2\right)^2}\delta^2\right) \\ &\approx \mathrm{e}^{-2\Delta_2\delta_\tau}\left(\mathrm{e}^{-2\Delta_1\delta_\tau} f_0\delta^2 + f_1\delta^2\right) \\ &= \mathrm{e}^{-2\delta_\tau(\Delta_1+\Delta_2)} f_0\delta^2 + \mathrm{e}^{-2\delta_\tau(\Delta_2)} f_1\delta^2 \end{aligned} \tag{S26}$$

Iterating this process, we get the following upper-bound for the final fidelity:

$$1 - F_N \leq \sum_{s=1}^{N} \mathrm{e}^{-2\delta_\tau \sum_{i=s}^{N} \Delta_i} f_{s-1} \delta^2 = \frac{1}{N^2} \sum_{s=1}^{N} \mathrm{e}^{-2\delta_\tau \sum_{i=s}^{N} \Delta_i} f_{s-1} \ . \tag{S27}$$

Equation (S27) can be interpreted as follows. At each time step, an error proportional to the fidelity susceptibility of the ground state path is accumulated. This error is exponentially damped down during all the remaining evolution at a rate proportional to the instantaneous first energy gap.

Now, we recall that by construction, the operator $\hat{H}_s$ can be written in block diagonal form, where each block acts on a connected subgraph, i.e., on a single equivalence class. The dynamic is thus restricted to the subspace spanned by the equivalence class of the input word $\tilde{\omega}$. Thus, given the input word $\tilde{\omega}$, the fidelity susceptibility refers to the path of ground states in the corresponding sector of the Hilbert space, as well as the first energy gap.

Computational complexity and final energy gap

Equation (S27) is a bound that depends on the specific path of ground states in the exam, and, ultimately, on the input state $|\tilde{\omega}\rangle$. A less strong bound can be derived that does not depend on $\tilde{\omega}$ but only on the first energy gap of the Laplacian in the block where $\tilde{\omega}$ belongs. In the following, we call this gap the final gap $\Delta_{\tilde{\omega}}$.

First of all, we observe that in the last part of the dynamics, the accumulated error is damped at a rate that only depends on the gap of the Hamiltonian for $s \approx N$. Since the Hamiltonian norm is bounded, this gap can not deviate too much from $\Delta_{\tilde{\omega}}$. This observation can be placed on a more rigorous mathematical footing using Weyl's perturbation theorem (*85*), which establishes that the rate of change of eigenvalues is bounded by the operator norm of the variation of the Hamiltonian as

$$|E_{i,s} - E_{i,N}| \leq ||H_s - H_N||_{\mathrm{op}} = \left\| \frac{N-s}{N} \left(\hat{\mathcal{L}}_S + |\tilde{\omega}\rangle\langle\tilde{\omega}| \right) \right\|_{\mathrm{op}} = \frac{N-s}{N} \left\| \hat{\mathcal{L}}_S + |\tilde{\omega}\rangle\langle\tilde{\omega}| \right\|_{\mathrm{op}} \ . \tag{S28}$$

We can upper-bound the Hamiltonian norm as

$$||\hat{\mathcal{L}}_S + |\tilde{\omega}\rangle\langle\tilde{\omega}|||_{\mathrm{op}} \leq \sum_r ||\hat{r}^2 - r||_{\mathrm{op}} + |||\tilde{\omega}\rangle\langle\tilde{\omega}|||_{\mathrm{op}} \leq 2n_r + 1 \tag{S29}$$

where we used the triangular inequality for the operator norm, n_r is the number of operators $\hat{r}^2 - \hat{r}$

in the Laplacian, and each operator $\hat{r}^2 - \hat{r}$ has norm 2. Thus, Eq. (S28) becomes

$$|E_{i,s} - E_{i,N}| \le \frac{N-s}{N}(2n_r + 1) , \tag{S30}$$

which implies

$$\Delta_s > \Delta_{\tilde{\omega}} - \frac{N-s}{N}(4n_r + 2) . \tag{S31}$$

Let s_* be the minimum value of s for which the last expression is positive, that is

$$s_* = N - \frac{N\Delta_{\tilde{\omega}}}{4n_r + 2} . \tag{S32}$$

For $s < s_*$ we have

$$\mathrm{e}^{-2\delta_\tau \sum_{i=s}^N \Delta_i} \le \mathrm{e}^{-2\delta_\tau \sum_{i=s_*}^N \Delta_i} \le \mathrm{e}^{-2\delta_\tau \sum_{i=s_*}^N \left(\Delta_{\tilde{\omega}} - \frac{N-i}{N}(4n_r+2)\right)} = \mathrm{e}^{-N\frac{\delta_\tau \Delta_{\tilde{\omega}}^2}{4n_r+2}} . \tag{S33}$$

and for $s \ge s*$

$$\mathrm{e}^{-2\delta_\tau \sum_{i=s}^N \Delta_i} \le \mathrm{e}^{-2\delta_\tau \sum_{i=s}^N \left(\Delta_{\tilde{\omega}} - \frac{N-i}{N}(4n_r+2)\right)} = \mathrm{e}^{-2\delta_\tau\left((N-s)\Delta_{\tilde{\omega}} - \frac{(N-s)^2}{2N}(4n_r+2)\right)} . \tag{S34}$$

We substitute these two bounds in Eq. (S27) to obtain

$$1 - F_N \le \frac{1}{N^2}\left(\sum_{s=1}^{s_*-1} f_{s-1}\mathrm{e}^{-N\frac{\delta_\tau \Delta_{\tilde{\omega}}^2}{4n_r+2}} + \sum_{s=s_*}^{N} f_{s-1}\mathrm{e}^{-2\delta_\tau\left((N-s)\Delta_{\tilde{\omega}} - \frac{(N-s)^2}{2N}(4n_r+2)\right)}\right) \tag{S35}$$

For large N, the second summation can be written as an integral. With a change of variable, we obtain

$$1 - F_N \le \frac{f_{\mathrm{avg}}}{N}\mathrm{e}^{-N\frac{\delta_\tau \Delta_{\tilde{\omega}}^2}{4n_r+2}} + \frac{f_f}{N}\int_0^1 \mathrm{e}^{-\frac{2\delta_\tau N\Delta_{\tilde{\omega}}}{4n_r+2}(x-x^2)}dx . \tag{S36}$$

For large N, we can use Laplace's method for approximating the integral by expanding the exponent around the endpoints of the domain, thus obtaining:

$$1 - F_N \le \frac{f_{\mathrm{avg}}}{N}\mathrm{e}^{-\frac{N\delta_\tau \Delta_{\tilde{\omega}}^2}{4n_r+2}} + \frac{f_f}{N}\frac{4n_r + 2}{N\delta_\tau \Delta} . \tag{S37}$$

Now we bound the fidelity susceptibility at the end of the process with respect to the final energy gap (*84*) as

$$f_s = \sum_{n\neq 0}\frac{|\langle n_s|\partial_s H_s|0_s\rangle|^2}{(E_{n,s} - E_{0,s})^2} \le \frac{||\partial_s H_s||_{\mathrm{op}}}{\Delta_{\tilde{\omega}}^2}, \tag{S38}$$

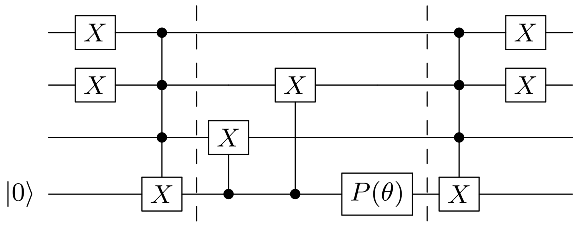

Figure S1: **Circuit implementation of** $e^{-i\theta|010\rangle\langle 001|}$**.** In the first part of the circuit, we flip the ancilla only if the state is $|001\rangle$. In the second part, we map $|001\rangle$ to $|010\rangle$ and add a global phase if the ancilla is in the state $|1\rangle$. Finally, we uncompute the ancilla.

which, in our case, considering the bound on the Laplacian norm in Eq. (S29), becomes

$$f_s \leq \frac{4n_r + 2}{\Delta_{\tilde{\omega}}^2}. \tag{S39}$$

Replacing the bound on the final fidelity in Eq. (S37) we obtain

$$1 - F_N \leq \frac{f_{\text{avg}}}{N} e^{-\frac{N\delta_\tau \Delta_{\tilde{\omega}}^2}{4n_r+2}} + \frac{(4n_r + 2)^2}{N^2 \delta_\tau \Delta^3} \,. \tag{S40}$$

Then, the number of time steps needed to get a final infidelity $1 - F_N \leq \epsilon$ is

$$N = O\left(\frac{n_r}{\Delta_{\tilde{\omega}}^2} \log\left(\frac{f_{\text{avg}}}{\epsilon}\right)\right) + O\left(\sqrt{\frac{n_r^2}{\epsilon \delta_\tau \Delta_{\tilde{\omega}}^3}}\right) \,. \tag{S41}$$

Thus, when the number of rules in the rewriting system grows polynomially, the computational complexity scales polynomially with the inverse of the final minimum energy gap. This result holds even if the fidelity susceptibility diverges exponentially during the evolution, as a consequence of an exponentially closing gap. By contrast, this advantage is lost in real-time quantum annealing: due to the unitary nature of the dynamics, errors accumulated at intermediate times cannot be dissipated at the end of the process.

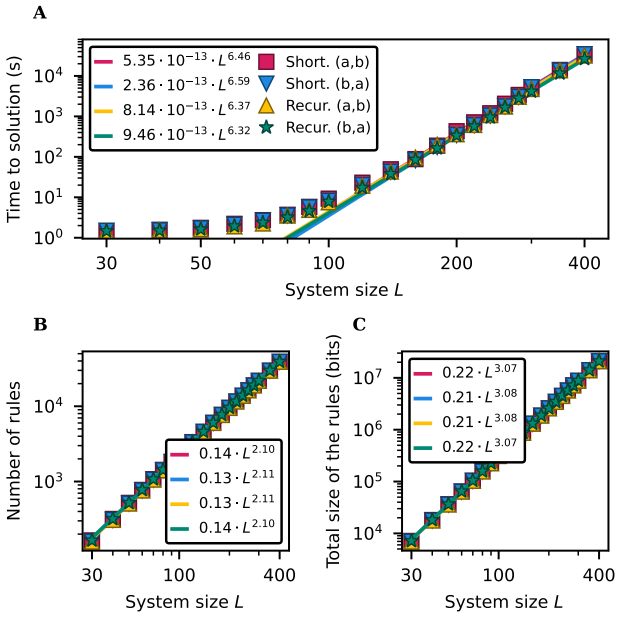

Figure S2: **Computational cost of the Knuth-Bendix algorithm as a function of the string size L for different orderings.** *Panel A:* Computational time required to construct a confluent string rewriting system, along with the corresponding polynomial fit for $L \geq 150$. *Panel B:* Number of rules generated in the confluent string rewriting system, together with the corresponding polynomial fit. *Panel C:* Total size of the rules in the confluent string rewriting system, along with the corresponding polynomial fit.

REFERENCES

1. J. W. Klop, M. Bezem, R. C. D. Vrijer, *Term Rewriting Systems* (Cambridge Univ. Press, 2001).

2. F. Baader, T. Nipkow, *Term rewriting and all that* (Cambridge Univ. Press, 2012).

3. D. A. Plaisted, Equational reasoning and term rewriting systems. *Handb. Logic Artif. Intell. Logic Program.* **1**, 273–364 (1993).

4. C. C. Sims, *Computation with Finitely Presented Groups* (Encyclopedia of Mathematics and its Applications, Cambridge Univ. Press, 2010).

5. J. Hsiang, Refutational theorem proving using term-rewriting systems. *Artif Intell* **25**, 255–300 (1985).

6. A. Clément, N. Heurtel, S. Mansfield, S. Perdrix, B. Valiron, A Complete Equational Theory for Quantum Circuits, in *2023 38th Annual ACM/IEEE Symposium on Logic in Computer Science (LICS)* (IEEE, 2023), pp. 1–13.

7. R. Wille, D. Große, D. M. Miller, R. Drechsler, Equivalence checking of reversible circuits, in *2009 39th International Symposium on Multiple-Valued Logic* (IEEE, 2009), pp. 324–330.

8. L. Burgholzer, R. Wille, Advanced equivalence checking for quantum circuits. *IEEE Trans. Comput. Aided Design Integr. Circuits Syst.* **40**, 1810–1824 (2021).

9. J. Kusyk, S. M. Saeed, M. U. Uyar, Survey on quantum circuit compilation for noisy intermediate-scale quantum computers: Artificial intelligence to heuristics. *IEEE Trans. Quantum Eng.* **2**, 1–16 (2021).

10. R. Duncan, A. Kissinger, S. Perdrix, J. van de Wetering, Graph-theoretic simplification of quantum circuits with the ZX-calculus. *Quantum* **4**, 279 (2020).

11. N. Chomsky, *Syntactic structures* (Mouton de Gruyter, 2002).

12. J. E. Hopcroft, R. Motwani, J. D. Ullman, *Introduction to Automata Theory, Languages, and Computation* (Addison-Wesley Longman Publishing Co. Inc., ed. 3, 2006).

13. A. V. Aho, M. S. Lam, R. Sethi, J. D. Ullman, *Compilers: Principles, Techniques, and Tools* (Addison-Wesley Longman Publishing Co. Inc., ed. 2, 2006).

14. J. Kieffer, E.-H. Yang, Grammar-based codes: A new class of universal lossless source codes. *IEEE Trans. Inf. Theory* **46**, 737–754 (2000).

15. D. B. Searls, The language of genes. *Nature* **420**, 211–217 (2002).

16. D. B. Searls, A primer in macromolecular linguistics. *Biopolymers* **99**, 203–217 (2013).

17. R. Stoyan, V. Strehl, Enumeration of Hamiltonian circuits in rectangular grids. *J. Comb. Math. Comb. Comput.* **21**, 109–128 (1996).

18. W. W. Boone, The word problem. *Proc. Natl. Acad. Sci. U.S.A.* **44**, 1061–1065 (1958).

19. G. Sénizergues, The equivalence problem for deterministic pushdown automata is decidable, in *Automata, Languages and Programming*, P. Degano, R. Gorrieri, A. Marchetti-Spaccamela, Eds. (Springer Berlin Heidelberg, 1997), pp. 671–681.

20. O. Bodroza-Pantic, B. Pantic, I. Pantic, M. Bodroza-Solarov, Enumeration of Hamiltonian cycles in some grid graphs. *MATCH Commun. Math. Comput. Chem* **70**, 181–204 (2013).

21. C. Vanderzande, *Lattice Models of Polymers* (Cambridge Lecture Notes in Physics, Cambridge Univ. Press, 2010).

22. G. Y. Vichniac, Simulating physics with cellular automata. *Physica D* **10**, 96–116 (1984).

23. H. Ellegren, N. Galtier, Determinants of genetic diversity. *Nat. Rev. Genet.* **17**, 422–433 (2016).

24. M. A. Nielsen, I. L. Chuang, *Quantum Computation and Quantum Information* (Cambridge Univ. Press, ed. 10, 2011).

25. T. D. Ladd, F. Jelezko, R. Laflamme, Y. Nakamura, C. Monroe, J. L. O'Brien, Quantum computers. *Nature* **464**, 45–53 (2010).

26. A. Montanaro, Quantum algorithms: An overview. *npj Quantum Inform.* **2**, 15023 (2016).

27. D. E. Knuth, P. B. Bendix, *Simple Word Problems in Universal Algebras* (Springer Berlin Heidelberg, 1983), pp. 342–376.

28. F. R. K. Chung, Spectral Graph Theory (American Mathematical Society, 1997).

29. T. Kadowaki, H. Nishimori, Quantum annealing in the transverse Ising model. *Phys. Rev. E* **58**, 5355–5363 (1998).

30. E. Farhi, J. Goldstone, S. Gutmann, J. Lapan, A. Lundgren, D. Preda, A quantum adiabatic evolution algorithm applied to random instances of an NP-complete problem. *Science* **292**, 472–475 (2001).

31. G. E. Santoro, R. Martonak, E. Tosatti, R. Car, Theory of quantum annealing of an Ising spin glass. *Science* **295**, 2427–2430 (2002).

32. T. Albash, D. A. Lidar, Adiabatic quantum computation. *Rev. Mod. Phys.* **90**, 015002 (2018).

33. J. Werschnik, E. K. U. Gross, Quantum optimal control theory. *J. Phys. B At. Mol. Opt. Phys.* **40**, R175–R211 (2007).

34. P. Doria, T. Calarco, S. Montangero, Optimal control technique for many-body quantum dynamics. *Phys. Rev. Lett.* **106**, 190501 (2011).

35. T. Caneva, T. Calarco, S. Montangero, Chopped random-basis quantum optimization. *Phys. Rev. A* **84**, 022326 (2011).

36. E. Farhi, J. Goldstone, S. Gutmann, A quantum approximate optimization algorithm. arXiv:1411.4028 [quant-ph] (2014).

37. E. Farhi, A. W. Harrow, Quantum supremacy through the quantum approximate optimization algorithm. arXiv:1602.07674 [quant-ph] (2019).

38. K. Blekos, D. Brand, A. Ceschini, C. H. Chou, R. H. Li, K. Pandya, A. Summer, A review on quantum approximate optimization algorithm and its variants. *Phys. Rep.* **1068**, 1–66 (2024).

39. S. McArdle, T. Jones, S. Endo, Y. Li, S. C. Benjamin, X. Yuan, Variational ansatz-based quantum simulation of imaginary time evolution. *npj Quantum Inform.* **5**, 75 (2019).

40. M. Motta, C. Sun, A. T. K. Tan, M. J. O'Rourke, E. Ye, A. J. Minnich, F. G. S. L. Brandão, G. K. L. Chan, Determining eigenstates and thermal states on a quantum computer using quantum imaginary time evolution. *Nat. Phys.* **16**, 205–210 (2020).

41. H. Kamakari, S.-N. Sun, M. Motta, A. J. Minnich, Digital quantum simulation of open quantum systems using quantum imaginary–time evolution. *PRX Quantum* **3**, 010320 (2022).

42. A. Abbas, A. Ambainis, B. Augustino, A. Bärtschi, H. Buhrman, C. Coffrin, G. Cortiana, V. Dunjko, D. J. Egger, B. G. Elmegreen, N. Franco, F. Fratini, B. Fuller, J. Gacon, C. Gonciulea, S. Gribling, S. Gupta, S. Hadfield, R. Heese, G. Kircher, T. Kleinert, T. Koch, G. Korpas, S. Lenk, J. Marecek, V. Markov, G. Mazzola, S. Mensa, N. Mohseni, G. Nannicini, C. O'Meara, E. P. Tapia, S. Pokutta, M. Proissl, P. Rebentrost, E. Sahin, B. C. B. Symons, S. Tornow, V. Valls, S. Woerner, M. L. Wolf-Bauwens, J. Yard, S. Yarkoni, D. Zechiel, S. Zhuk, C. Zoufal, Challenges and opportunities in quantum optimization. *Nat. Rev. Phys.* **6**, 718–735 (2024).

43. H. Buhrman, R. Cleve, J. Watrous, R. de Wolf, Quantum fingerprinting. *Phys. Rev. Lett.* **87**, 167902 (2001).

44. J. Haegeman, C. Lubich, I. Oseledets, B. Vandereycken, F. Verstraete, Unifying time evolution and optimization with matrix product states. *Phys. Rev. B* **94**, 165116 (2016).

45. U. Schollwöck, The density-matrix renormalization group. *Rev. Mod. Phys.* **77**, 259–315 (2005).

46. U. Schollwöck, The density-matrix renormalization group in the age of matrix product states. *Ann. Phys.* **326**, 96–192 (2011).

47. S. Montangero, *Introduction to Tensor Network Methods* (Springer, 2018).

48. P. Silvi, F. Tschirsich, M. Gerster, J. Jünemann, D. Jaschke, M. Rizzi, S. Montangero, The tensor networks anthology: Simulation techniques for many-body quantum lattice systems. *SciPost Phys. Lect. Ther. Notes* 10.21468/SciPostPhysLectNotes.8 (2019).

49. G. Evenbly, G. Vidal, Tensor network states and geometry. *J. Stat. Phys.* **145**, 891–918 (2011).

50. D. Rattacaso, G. Passarelli, A. Russomanno, P. Lucignano, G. E. Santoro, R. Fazio, Parent hamiltonian reconstruction via inverse quantum annealing. *Phys. Rev. Lett.* **132**, 160401 (2024).

51. X. Turkeshi, T. Mendes-Santos, G. Giudici, M. Dalmonte, Entanglement-guided search for parent Hamiltonians. *Phys. Rev. Lett.* **122**, 150606 (2019).

52. C. Fernández-González, N. Schuch, M. M. Wolf, J. I. Cirac, D. Pérez-García, Frustration free gapless Hamiltonians for matrix product states. *Commun. Math. Phys.* **333**, 299–333 (2015).

53. A. del Campo, Shortcuts to adiabaticity by counterdiabatic driving. *Phys. Rev. Lett.* **111**, 100502 (2013).

54. D. Guéry-Odelin, A. Ruschhaupt, A. Kiely, E. Torrontegui, S. Martínez-Garaot, J. G. Muga, Shortcuts to adiabaticity: Concepts, methods, and applications. *Rev. Mod. Phys.* **91**, 045001 (2019).

55. T. Zanca, G. E. Santoro, Quantum annealing speedup over simulated annealing on random Isig chains. *Phys. Rev. B* **93**, 224431 (2016).

56. R. Islam, R. Ma, P. M. Preiss, M. Eric Tai, A. Lukin, M. Rispoli, M. Greiner, Measuring entanglement entropy in a quantum many-body system. *Nature* **528**, 77–83 (2015).

57. J. Eisert, M. Cramer, M. B. Plenio, Colloquium: Area laws for the entanglement entropy. *Rev. Mod. Phys.* **82**, 277–306 (2010).

58. M. Ballarin, P. Silvi, S. Montangero, D. Jaschke, Optimal sampling of tensor networks targeting wave function's fast decaying tails. *Quantum* **9**, 1714 (2025).

59. H. W. Lin, M. Tegmark, Critical behavior in physics and probabilistic formal languages. *Entropy* **19**, 299 (2017).

60. S. Lu, M. Kanász-Nagy, I. Kukuljan, J. I. Cirac, Tensor networks and efficient descriptions of classical data. *Phys. Rev. A* **111**, 032409 (2025).

61. A. J. Gallego, R. Orús, Language design as information renormalization. *SN Comput. Sci.* **3**, 140 (2022).

62. Y.-Y. Shi, L.-M. Duan, G. Vidal, Classical simulation of quantum many-body systems with a tree tensor network. *Phys. Rev. A* **74**, 022320 (2006).

63. P. Silvi, V. Giovannetti, S. Montangero, M. Rizzi, J. I. Cirac, R. Fazio, Homogeneous binary trees as ground states of quantum critical Hamiltonians. *Phys. Rev. A* **81**, 062335 (2010).

64. M. Gerster, P. Silvi, M. Rizzi, R. Fazio, T. Calarco, S. Montangero, Unconstrained tree tensor network: An adaptive gauge picture for enhanced performance. *Phys. Rev. B* **90**, 125154 (2014).

65. I. Arad, A. Kitaev, Z. Landau, U. Vazirani, An area law and sub-exponential algorithm for 1D systems. arXiv:1301.1162 [quant-ph] (2013).

66. T. Felser, S. Notarnicola, S. Montangero, Efficient tensor network ansatz for high-dimensional quantum many-body problems. *Phys. Rev. Lett.* **126**, 170603 (2021).

67. T. H. Cormen, C. E. Leiserson, R. L. Rivest, C. Stein, *Introduction to Algorithms* (The MIT Press, ed. 3, 2009).

68. J. D. Biamonte, J. Morton, J. Turner, Tensor network contractions for #SAT. *J. Stat. Phys.* **160**, 1389–1404 (2015).

69. S. Kourtis, C. Chamon, E. R. Mucciolo, A. E. Ruckenstein, Fast counting with tensor networks. *SciPost Phys.* **7**, 060 (2019).

70. N. Wiebe, A. Bocharov, P. Smolensky, M. Troyer, K. M. Svore, Quantum language processing. arXiv:1902.05162 [quant-ph] (2019).

71. B. Coecke, G. de Felice, K. Meichanetzidis, A. Toumi, Foundations for near-term quantum natural language processing. arXiv:2012.03755 [quant-ph] (2020).

72. D. Rattacaso, D. Jaschke, M. Ballarin, I. Siloi, S. Montangero, Quantum circuit compilation with quantum computers. arXiv:2408.00077 [quant-ph] (2024).

73. A. Barenco, C. H. Bennett, R. Cleve, D. P. DiVincenzo, N. Margolus, P. Shor, T. Sleator, J. A. Smolin, H. Weinfurter, Elementary gates for quantum computation. *Phys. Rev. A* **52**, 3457–3467 (1995).

74. M. Khazali, K. Mølmer, Fast multiqubit gates by adiabatic evolution in interacting excited-state manifolds of Rydberg atoms and superconducting circuits. *Phys. Rev. X* **10**, 021054 (2020).

75. GAP–Groups, Algorithms, and Programming, version 4.14.0 (2024); www.gap-system.org.

76. F. Baccari, D. Bacilieri, M. Ballarin, F. P. Barone, F. Campaioli, A. G. Catalano, G. Cataldi, A. Coppi, A. Costantini, A. Datta, A. De Girolamo, D. Jaschke, S. B. Kožić, G. Magnifico, C. Mordini, S. Montangero, S. Notarnicola, A. Pagano, L. Pavesic, D. Rattacaso, M. Rigobello, N. Reinić, S. Scarlatella, l. Siloi, P. Silvi, M. Tesoro, G. Torre, D. Wanisch, Quantum TEA: qtealeaves (Zenodo, 2025); https://doi.org/10.5281/zenodo.10498928.

77. D. Rattacaso, D. Jaschke, M. Ballarin, I. Siloi, S. Montangero, Complete code and data from: Quantum algorithms for equational reasoning (Zenodo, 2026) https://doi.org/10.5281/zenodo.19914630.

78. Y. Wang, Z. Hu, B. C. Sanders, S. Kais, Qudits and high-dimensional quantum computing. *Front. Phys.* **8**, 589504 (2020).

79. S. Lloyd, Universal quantum simulators. *Science* **273**, 1073–1078 (1996).

80. A. Montanaro, Quantum speedup of Monte Carlo methods. *Proc. A* **471**, 20150301 (2015).

81. H. Zankl, C. Sternagel, D. Hofbauer, A. Middeldorp, Finding and Certifying Loops, in *SOFSEM 2010: Theory and Practice of Computer Science*, J. van Leeuwen, A. Muscholl, D. Peleg, J. Pokorný, B. Rumpe, Eds. (Springer Berlin Heidelberg, 2010), pp. 755–766.

82. S. Chakraborty, K. S. Meel, M. Y. Vardi, *A scalable approximate model counter, in principles and practice of constraint programming*, C. Schulte, Ed. (Springer Berlin Heidelberg, 2013), pp. 200–216.

83. J. Kempe, A. Kitaev, O. Regev, The complexity of the local Hamiltonian problem. *SIAM J. Comput.* **35**, 1070–1097 (2006).

84. L. Campos Venuti, P. Zanardi, Quantum critical scaling of the geometric tensors. *Phys. Rev. Lett.* **99**, 095701 (2007).

85. R. Bhatia, *Matrix analysis, Graduate texts in mathematics* (Springer, 1997).
